# Integrating Local and Global Error Statistics for Multi-Scale RBF Network Training: An Assessment on Remote Sensing Data

**DOI:** 10.1371/journal.pone.0040093

**Published:** 2012-08-02

**Authors:** Giorgos Mountrakis, Wei Zhuang

**Affiliations:** Department of Environmental Resources Engineering, State University of New York College of Environmental Science and Forestry, Syracuse, New York, United States of America; Pacific Climate Impacts Consortium, Canada

## Abstract

**Background:**

This study discusses the theoretical underpinnings of a novel multi-scale radial basis function (MSRBF) neural network along with its application to classification and regression tasks in remote sensing. The novelty of the proposed MSRBF network relies on the integration of both local and global error statistics in the node selection process.

**Methodology and Principal Findings:**

The method was tested on a binary classification task, detection of impervious surfaces using a Landsat satellite image, and a regression problem, simulation of waveform LiDAR data. In the classification scenario, results indicate that the MSRBF is superior to existing radial basis function and back propagation neural networks in terms of obtained classification accuracy and training-testing consistency, especially for smaller datasets. The latter is especially important as reference data acquisition is always an issue in remote sensing applications. In the regression case, MSRBF provided improved accuracy and consistency when contrasted with a multi kernel RBF network.

**Conclusion and Significance:**

Results highlight the potential of a novel training methodology that is not restricted to a specific algorithmic type, therefore significantly advancing machine learning algorithms for classification and regression tasks. The MSRBF is expected to find numerous applications within and outside the remote sensing field.

## Introduction

Remote sensing has been recognized as a highly effective method for observing environmental changes at multiple temporal, spatial and spectral resolutions [1]. Data from various sensors has already been successful in extracting horizontal and vertical information of land cover and land use [2], [3], [4]. Numerous algorithms have either been developed or transitioned from other disciplines to assist for processing of remotely sensed data. Among other techniques, neural networks (NN) are frequently used in the analysis of remote sensing data since they do not require a specific distribution for the input data [5]. The Radial Basis Function (RBF) neural network is a type of feed-forward neural network that has attracted attention in remote sensing applications. For instance, the K-mean based RBF network was implemented for land cover classification [5]. It was proven difficult though to define in advance the number of centers for the K-mean method. A fuzzy mean method was applied in classifying IKONOS image, which calculated the RBF centers based on a triangular fuzzy partition of the input space [6], [7]. An orthogonal least square (OLS) learning algorithm was also used to find the parameters for the RBF nodes in the hidden layer in soil type classification task [8]. RBF networks have been compared to back propagation neural networks and probabilistic neural networks in a land cover classification problem, and obtained equal or better results [9], [10], [11], [12], [13]. RBF networks also have shown promising ability to classify multi-temporal imagery and update classification results using an incremental learning strategy [14], [15], [16], [17], [18]. Beyond classification tasks, the processing of a complex signal can be seen as a curve-fitting problem [19], leading to regression applications. For example, RBF networks have also been applied to Radar signals in order to derive biophysical parameters, such as snowfall, rainfall and wind speed [20], [21], [22], [23], [24], [25], [26].

### Kernel overlapping issue

The successful applicability of RBF networks mainly depends on two choices: the selection of centers and widths of the kernel functions, and magnitude assignments for data located within the overlapping area of the kernel functions [27]. The overlapping problem may be controlled through the selection of small kernel widths; however this limits the generalization ability of the network. Furthermore, RBF networks with large kernel widths may also lead to generalization errors if the overlapping area is large [17], [27], [28]. The novel RBF network proposed in this paper attempts to find the optimal balance between these two issues through multi-scale kernel width incorporation.

**Figure 1:**
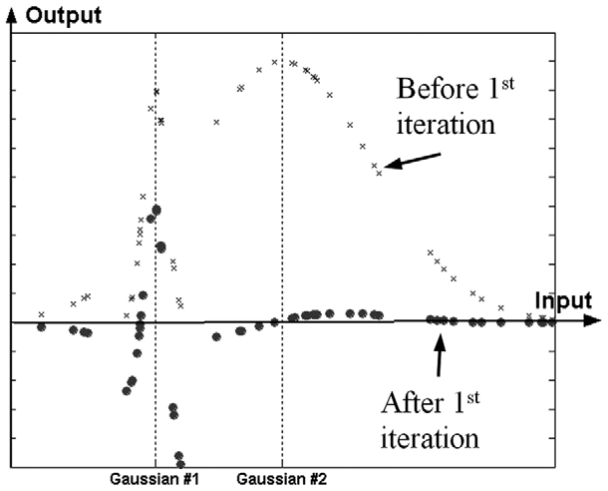
Activation function overlapping problem.

**Figure 2:**
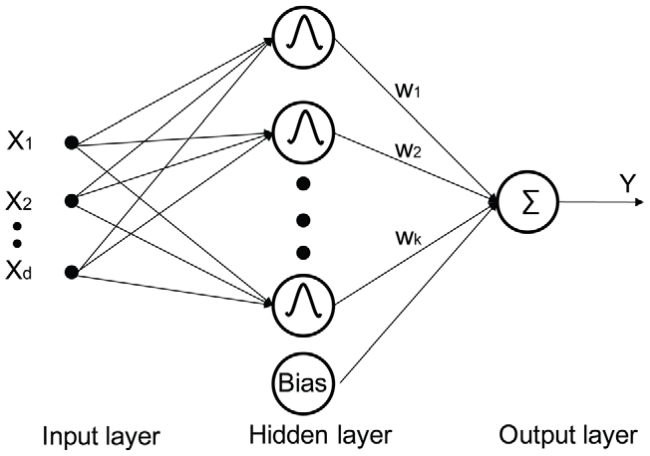
Conventional RBF architecture.

An example of the overlapping issue is investigated in Fig. 1. Assume an artificial dataset created from two Gaussian distributions, one with a large width and another with a much smaller one; also, assume the smaller pattern overlaps the larger one while the larger one is not active within the smaller pattern. The two patterns are depicted as *x*'s in Fig. 1. During the training process of a RBF network, the appropriate parameters (e.g. widths and center locations) for activation functions (AFs) should be selected for each node in the hidden layer. If AFs with small widths are chosen (e.g. widths close to the smaller width pattern), a higher number than two AFs would be necessary to capture the given dataset. On the other hand if AFs with large widths are implemented, the network would absorb the larger pattern but by doing so the structure of the smaller pattern would significantly change requiring a much higher number of nodes to capture it. Solid circles in Fig. 1 depict this issue after the first iteration chooses a large width AF and the selection of center location is affected by the smaller pattern on the left. The selection of large width AFs for this dataset would be typical during the RBF network training as the training process is guided to absorb the maximum possible signal from the first iteration.

**Figure 3:**
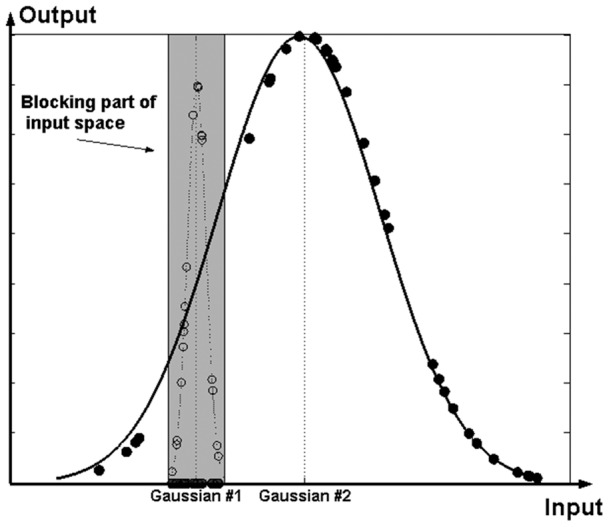
Blocking implementation example.

**Figure 4:**
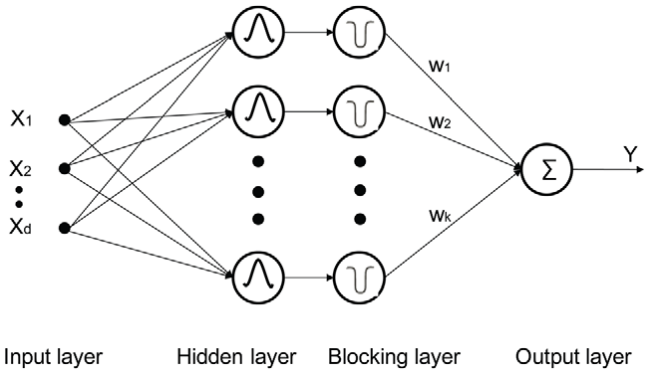
MSRBF architecture.

A number of algorithms have been developed to address the overlapping problem. Two learning schemes based on incremental training procedure were investigated in [29]: the first scheme suggested the addition of a new neuron to the entire structure and the second scheme examined the adjustment of weights connecting the hidden and output layers. Expectation-maximization and maximum likelihood algorithms were applied to split the overlapping areas into several sub-areas in an incremental probability RBF neural network so that the new split areas only contained the training samples with the same class [30]. In [31], [32], in order to decrease the number of nodes in a RBF networks, data within overlapping zone also involved in estimation of subsequent RBF function associated with nodes. By minimizing the responses variance of RBF functions from vectors that belong to the same class, the weights and parameters of RBF functions in a network can be updated in order to improve classification where significant class overlap exists [33]. In the aforementioned methods, the selection of centers and widths for the AFs was based on global error statistics, statistics that evaluate performance over the entire input space (e.g. Mean Square Error). There is a contradiction though as RBF networks by design are a neural network type that focuses more on local scale rather than global scale information since the AFs are bounded in the input space.

**Figure 5:**
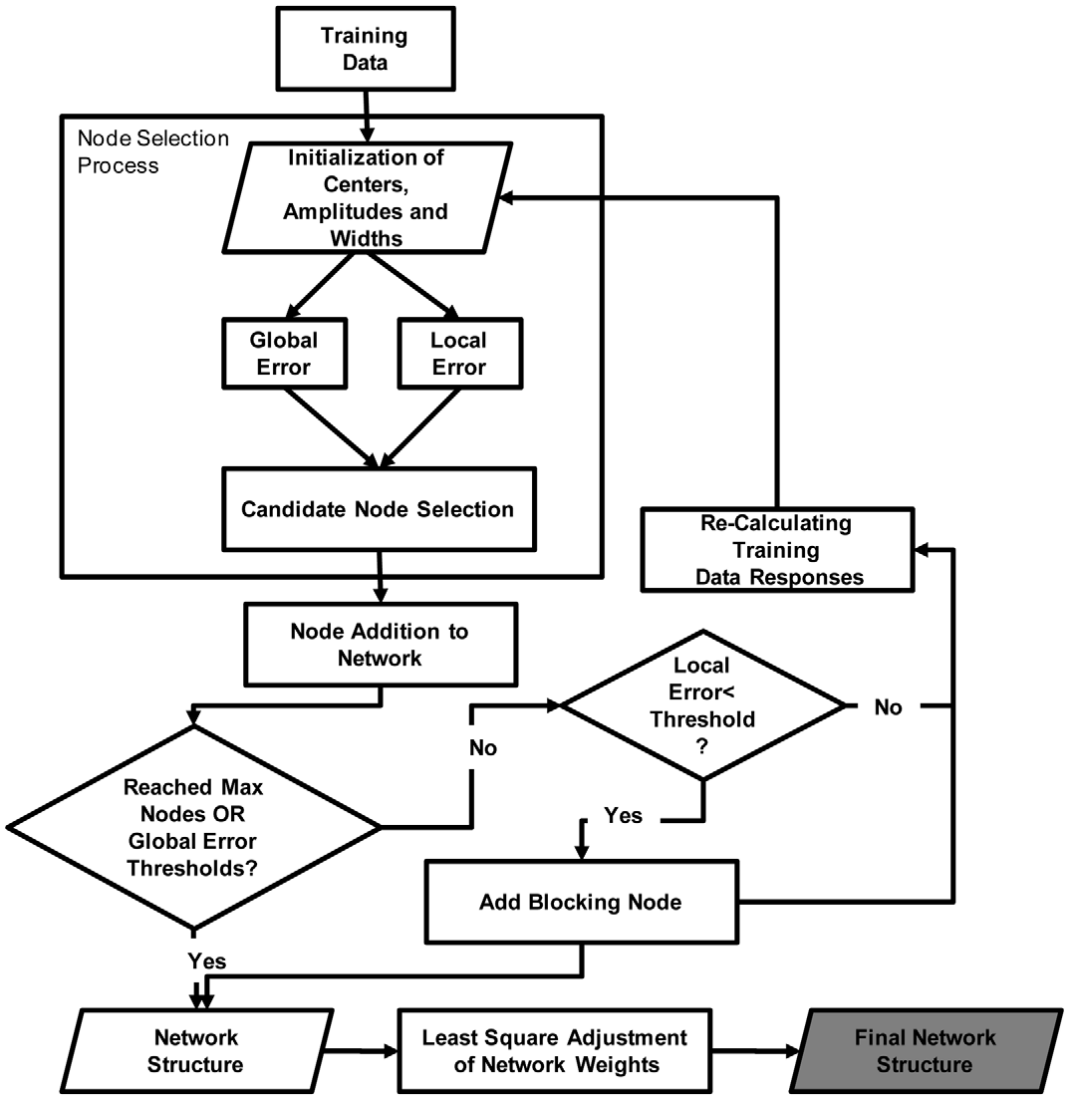
Schematic representation of training procedure.

**Figure 6:**
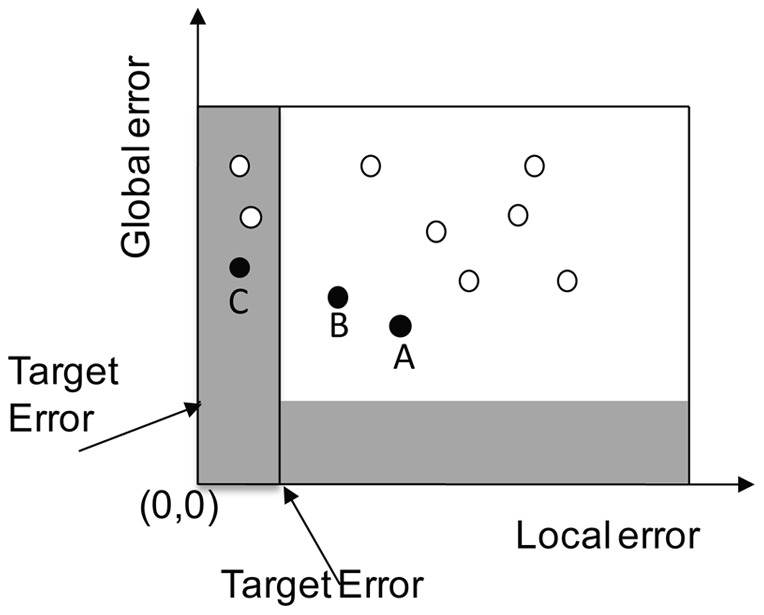
Node selection balancing local and global error absorption.

The premise of this paper is that both local and global behavior of a candidate neuron should be considered during the training process. A novel multi-scale RBF neural network [34] is proposed to minimize the effect of the overlapping problem. The changes from the traditional training process are discussed and the revised network architecture is presented. The method is evaluated on a binary classification task using Landsat imagery and a regression problem using waveform LiDAR data.

## Methods

The RBF network is a popular feed-forward neural network with applications in numerous fields, including image processing and analysis. RBF networks have three layers (shown in Fig. 2): an input layer, a hidden layer, and an output layer.

RBF training involves identification of the activation functions (AFs) embedded in the nodes of the hidden layer and the selection of weights connecting the hidden layer to the output layer. The selection of AFs, also known as kernel functions due to their localized influence, have been investigated for image classification purposes [5], [16], [35], [36]. The outputs of the RBF are linear combinations (shown in equation 1) of the responses from the kernel functions. The coefficients of the linear model are the weights linking the hidden layer and the output layer.
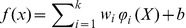
(1)


In the equation above, *f(X)* is the network output; *k* is the total number of nodes in the hidden layer; *w_i_* are the weights of the linear model; *b*-is the bias of the network. A least square solution is usually employed to solve this linear problem (shown in equation 1) by minimizing the squared norm of the residuals [36].

### Proposed MSRBF architecture

The underlying motivation behind the proposed MSRBF is to limit the initial greediness of typical RBF implementations, especially ones using kernels of multiple widths. As the example in Fig. 1 demonstrates, during training a global error minimization takes place in every node identification. There may be cases though where local signals mask larger scale signals leading to middle of the road solutions in typical RBF implementations.

**Figure 7:**
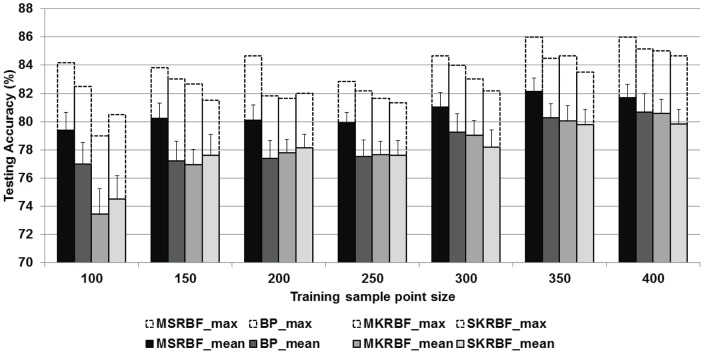
Accuracy comparison among four network types.

**Table 1:** Two-tailed Student T test between MSRBF and other algorithms.

Training size	t values			Training size	p values		
	BP	MKRBF	SKRBF		BP	MKRBF	SKRBF
100	8.50	21.58	16.63	100	<0.0001	<0.0001	<0.0001
150	12.15	15.32	9.79	150	<0.0001	<0.0001	<0.0001
200	11.74	11.18	9.65	200	<0.0001	<0.0001	<0.0001
250	11.40	13.11	12.70	250	<0.0001	<0.0001	<0.0001
300	7.69	9.40	12.63	300	<0.0001	<0.0001	<0.0001
350	9.45	10.06	11.64	350	<0.0001	<0.0001	<0.0001
400	4.61	5.83	9.26	400	<0.0001	<0.0001	<0.0001

Degree of Freedom is 98 (50*2−2), α = 0.05.

The proposed solution is to consider both local and global behavior in estimating the parameters of AF. Meanwhile, a blocking mechanism is established for parts of the input space that are successfully mapped, in essence parts within the receptive field of an AF where the local error is acceptable by training standards. The idea is that if successfully mapped areas are blocked from later node influence then the remaining patterns may be revealed and therefore captured leading to error minimization and node usage reduction in the hidden layer.

As Fig. 3 demonstrates if the small scale activation function is selected first (Gaussian #1) and these points (hollow circles) are excluded from further node training, then the global pattern will be revealed and easily captured in a subsequent node (Gaussian #2). Thus, a blocking layer is inserted between the hidden layer and the output layer in the conventional RBF network architecture aiming at selective blocking of the input space (Fig. 4).

**Figure 8:**
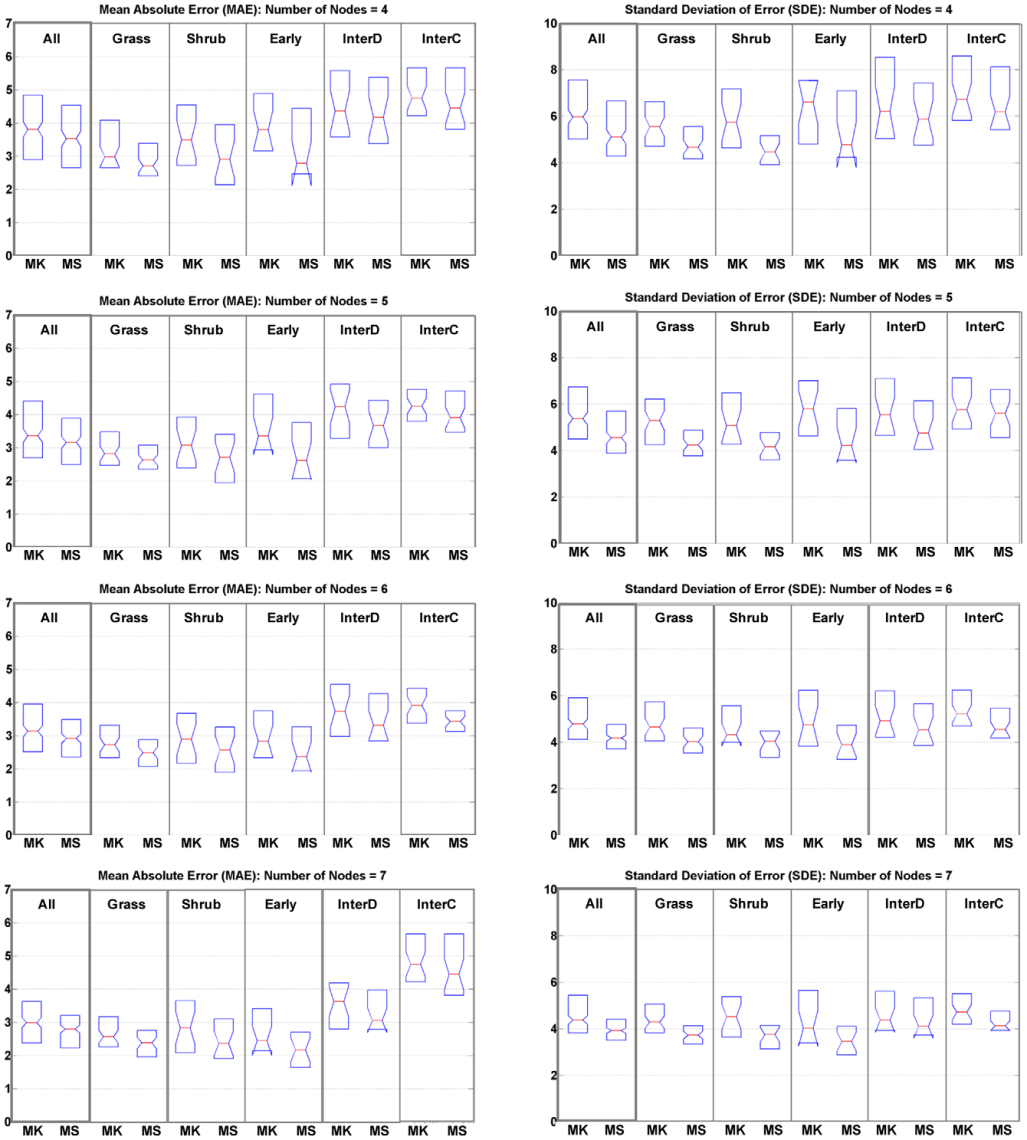
Comparison of MAE and SDE metrics for MK and MS RBFs.

**Table 2:** Mean comparison from ANOVA between MKRBF and MSRBF (All plots).

F test Hypothesis	Mean Comparison (*α = 0.05*)			
Nodes	4	5	6	7
MAE_MK≠MAE_MS	<0.01	<0.01	<0.001	<0.001
MASDE_MK≠MASDE_MS	<0.0001	<0.0001	<0.0001	<0.0001

The nodes in the blocking layer are exclusively associated one to one with the correspondent nodes in the hidden layer. A binary index is assigned to the connection to identify whether the blocking node is activated or not (1 for activated, 0 for inactivated). The outputs are calculated from the nodes in the blocking layer.

### Algorithmic Training Procedure

In order to facilitate further replication of the proposed method Fig. 5 identifies the major training steps of the proposed MSRBF:

Each candidate activation function (AF) was identified by parameters for the center, amplitude and widths. In the classification case, where a genetic algorithm was employed, random assignments for these parameters took place considering the boundaries from the training dataset. For the regression case, where no training optimization technique was incorporated, centers and associated amplitudes were selected from locations with large output values while widths followed an exhaustive search.

**Figure 9:**
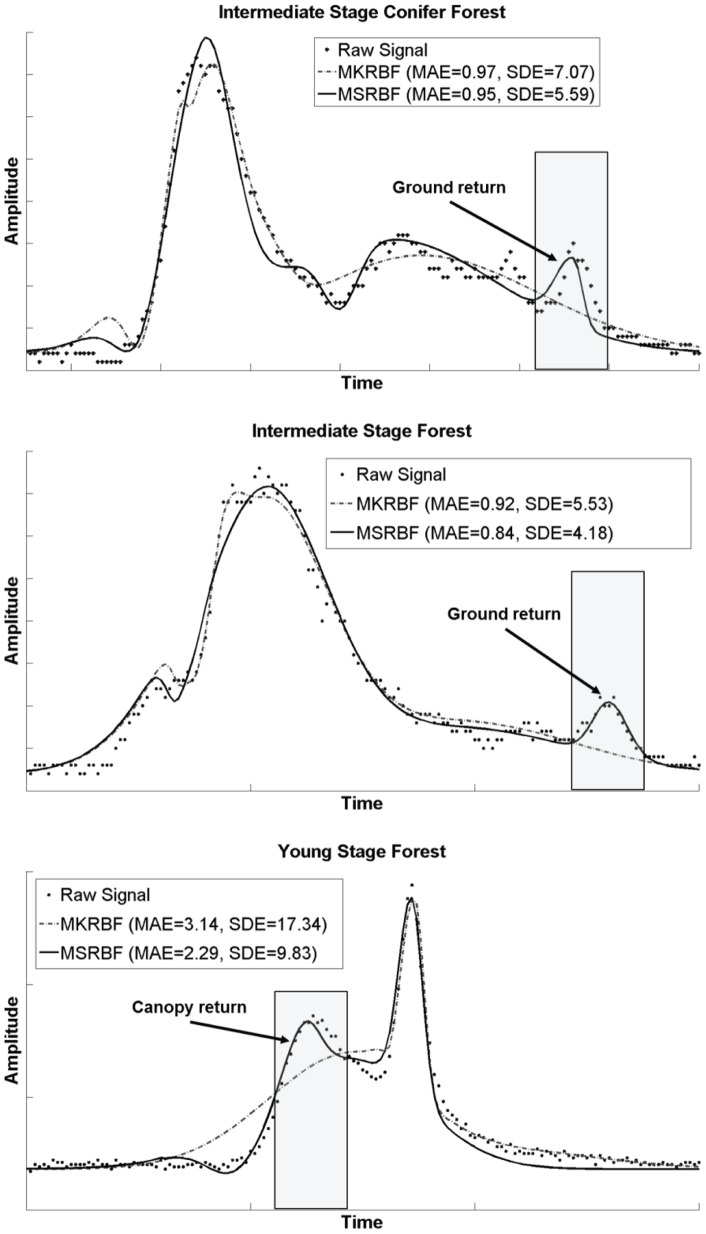
Contrasting MK and MS RBF fitting capabilities for biophysical feature extraction.

**Figure 10:**
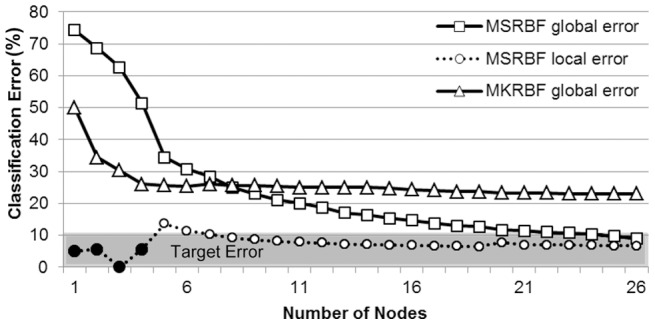
Contrasting MSRBF and MKRBF training progression.

**Figure 11:**
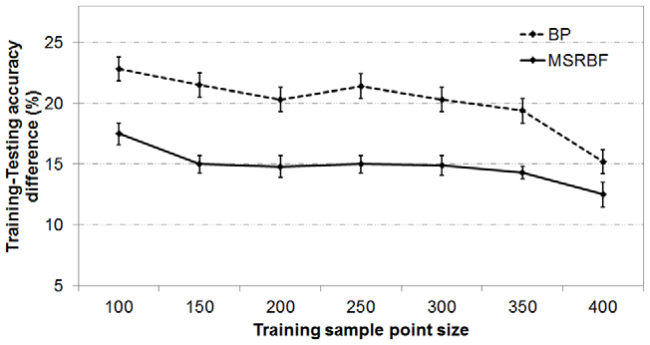
Mean and standard deviation on training-testing accuracy deviations.

After initialization of the parameters, global and local errors were calculated for each candidate AF. A node selection process followed balancing these two errors (see next section for details). After the addition of each node the training process examined whether a predetermined threshold of global error has been reached or whether the network had the maximum allowed nodes. If this was not the last node, the local error was contrasted with a predetermined error threshold. If it was sufficiently small a blocking node was added to the network structure associated with that node. At the last training step a least squares method was applied to fine tune the weights of each node.

### Node selection criteria balancing global and local errors

The major difference between the MSRBF training and typical RBF training is the incorporation of local error statistics in the node selection process. For each candidate activation function (AF) two evaluation metrics are produced: a Global Error (GE) and a Local Error (LE). In the case of a classification problem GE can be expressed as the ratio of the total number of misclassified points in the dataset to the number of all points in the dataset, while a LE is calculated as the GE but using exclusively points within the receptive field of the AF under consideration. In the case of a regression problem the GE can be based on the mean absolute error (MAE) of all dataset points, while the LE is the MAE exclusively from points within the AF receptive field.

A balancing act follows that takes into account both GE and LE. This is necessary as it expresses how aggressively local fits are pursued as opposed to global error absorption, especially in early node selection. The idea is that AFs with good local behavior are selected first to reveal larger scale signal(s). However, the AFs with good local behavior should also absorb a reasonable amount of the global signal to avoid significant increase in nodes. For example, the AF selection process should avoid fitting a single point of small magnitude, where the local error indeed will be minimal but undesirably this node would also have insignificant contribution to the overall solution.

Procedurally, GE and LE are first computed on all candidate AFs. At the next step a weighted sum of GE and LE is calculated for the selection of potential nodes, shown in equation.



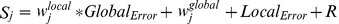
(2)where, *R* is an additional user-defined condition for the selections of nodes, such as the number of points located within a Gaussian function; *S_j_* is the weighted sum of AF candidate *j*, and *w_j_* denotes the local and global weights with 
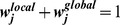
. The AF with the lowest weighted sum is selected. The influence of the local error is expressed through the local weight and it is a function of the following:
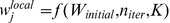
(3)where 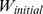 is the initial local weight value (0–1); 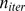
is the number of current iteration and K is the maximum total number of nodes allowed in the network (20–40 for classification case and 4–7 for regression case). In future implementations equations (2) and (3) can be replaced by other functions expressing a different global and local error tradeoff.

A visual example of AF winner selection is shown in Fig. 6. The circles represent the global and local errors for each AF candidate during a single iteration. Compared to the conventional node selection criterion, which only considers the global error, the proposed selection criterion also takes into account the local error. For example, if the conventional selection criterion is employed, the AF candidate A would be the winning choice among those candidates, since its global error is the smallest. On the other hand, the local error selection criterion may choose AF candidate C as the winning node since it minimizes error within the AF's receptive field. In most cases a balancing act would take place where both local and global errors are reduced, for example leading to the selection of candidate B.

The relative local and global weights (equation 2) provide an axes scaling mechanism in the above graphical representation and express this balancing act. Two additional points can be made from that graph. First, if a point existed with GE smaller than the Target Error (horizontal shaded area in the graph) then that would be automatically selected and iterations would end. Second, if the selected winner has LE smaller that the Target Error, a blocking mechanism is initiated since that neighborhood is successfully mapped and subsequent nodes should not interfere with that. This blocking mechanism links back to the formulation of matrix B in equations 5 and 6.

### Mathematical Solution

Matrices are typically used to represent responses of each node: assuming *k* nodes were used in the network and *n* was the number of sample points (i.e. input patterns), the following matrices can be derived:

Matrix Φ (n×k). In this matrix, the responses of sample points to every node in the hidden layer are recorded. The elements in each row represent the responses of a sample point to all nodes in the hidden layer; whereas the elements in the column signify all sample points' responses to a single node. This matrix is the core of every RBF network.
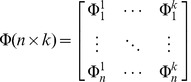
(4)


Matrix B (n×k). The dimensions of B are the same as Φ. This matrix permits or prevents the signal of a specific node to propagate to the final solution depending on the input pattern location in the input space and is expressed through the blocking function identification in the training process. Values of 1 allow propagation whereas 0 values do not. Each row corresponds to the blocking of a specific pattern to all nodes. Each column reflects the result of a specific blocking function to the input set.
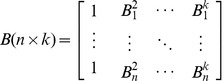
(5)


It is important to clarify the purpose of a blocking function, which is to “secure” that underlying neighborhood for all following iterations. A blocking function that is caused by node j will not have any effect on itself but will interfere with all the following nodes (j+1,…,k). In other words it will block subsequent nodes to interfere with that portion of the input space since it has already been modeled with sufficient accuracy. The first column of this blocking matrix is populated by 1s as no prior AF has been selected. The blocking of the chosen functions is such that their accumulative action is calculated by multiplication:
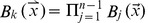
(6)


Matrix H (n×k). Due to the blocking layer insertion, the weights which link the hidden layer to the output layer now connect the blocking layer with the output layer. A recalculation of the weights is needed by the least squares solution. Final responses of the sample points to all the nodes can be calculated by an element-by-element product of Φ (equation 4) and *B* (equation 5), denoted as *H*.

Weights connecting the blocking layer and the output layer can be calculated by the typical equation (7):
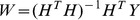
(7)where *W* is the weight vectors of all nodes in the hidden layer, and *Y* is the output values of the sample points.

To summarize, the MSRBF mathematical solution is similar to a typical RBF with the distinction of an elegant modification to the non-linear layer calculation that supports local neighborhood blocking.

## Results

Two general experiments took place using remotely sensed data, the first assessing the algorithm on a binary classification task using Landsat data and the second on a regression task using waveform LiDAR signals.

### Impervious classification example using Landsat data

Remote sensing has been extensively applied on monitoring impervious surfaces, the man-made structures constructed with impenetrable materials, such as roads, buildings and parking lots. Unfortunately, the spectral reflectance of impervious and non-impervious surfaces may often be similar, for example bare soil may be confused with concrete buildings [37]. Numerous approaches have been developed for impervious surface monitoring. For example, regression models targeted discovery of certain relationships between land cover types and sensor data [38], [39]. Linear Spectral Mixture Analysis (LSMA) was applied to map the subpixel land cover in medium resolution imagery [37], [40], [41], such as TM, ETM+ and ASTER. Moreover, the implementation of decision tree for impervious surfaces classification can also be found in [42], [43], [44]. As artificial neural networks can handle nonlinear relationships and make no assumptions for the data distribution [45], they also performed well in the classification of the impervious surfaces classification [46], [47], [48], [49], [50]. A recent review on impervious surface detection is available in [51]. However, limited research exists using RBF neural networks for impervious surface detection.

#### Landsat Data

A subset of a Landsat 7 Enhanced Thematic Mapper Plus (ETM+) image acquired on 18 April, 2006 with a size of 152×150 pixels and spatial resolution of 30 m was selected for this research. Principle component analysis was applied on the six bands, including blue, green, red, near IR and two mid IR bands and the first three components were used in the experiment. The variance explained in the three components was larger than 99%. A reference dataset depicting binary classes (impervious and non-impervious surface) was derived from manual interpretation of aerial digital orthophoto quarter quads imagery with spatial resolution of 2 m, also acquired in 2006. The reference dataset from the aerial image was resampled to the same pixel size of ETM+. If any proportion of a resampled pixel was occupied by impervious surface, this pixel was assigned to the impervious surface class. This strict binary threshold process was followed to increase the difficulty of the classification task and allow algorithmic assessment under a difficult but typical scenario. Furthermore, the produced binary classification is not expected to be a final product; instead it acts as an intermediate filtering for selective application of subpixel algorithms. Such intermediate binary filtering products are necessary as past impervious subpixel analysis has showed significant overestimation of imperviousness, especially in rural areas [42], [52]. Training and testing datasets of two classes were sampled using a stratified random strategy with equal number of points for both impervious and non-impervious classes. The two classes were presented as 1 and -1 outputs respectively. Furthermore, in order to assess algorithmic performance with respect to the size of the training dataset, different sample sizes were collected.

#### Landsat Experimental Setup

In order to accelerate MSRBF training for the classification task a genetic algorithm was incorporated to assist with activation function (AF) selection. Furthermore, a criterion was added to avoid overfitting expressed through the minimum number of points within the receptive field of an AF. Specifically, a criterion that incorporates the importance of the number of training points within the blocked neighborhood is added to the equation (2) when the local error is smaller than the Target Error (a predefined target error for successful training):
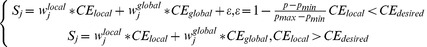
(8)


In the classification case, the local behavior is more important, since the cluster from a local Gaussian may represent a possible class member. Therefore, high priority needs to be given to local accuracy. The equation for calculating the local weight is shown below:



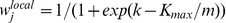
(9)where *k i*s the current number of nodes (i.e. iteration number), *K_max_* is the predetermined maximum node number for the MSRBF, and *m* is a user-defined parameter that controls the decrease rate for the local weight. Full details are provided in Text S1. To evaluate the effectiveness of the MSRBF network, a classification accuracy comparison between several algorithms was carried out, namely a Back-Propagation (BP), a single kernel RBF (SKRBF) and a multi-kernel RBF (MKRBF) neural network. The SKRBF was based on the built-in Matlab code and employed the same width for all kernel functions. The BP also used Matlab's built-in functions with the default Levenberg-Marquardt algorithm. The MKRBF was custom coded with similar properties as the proposed MSRBF but with the exception of using local errors in the node selection process. The BP network allowed evaluation of benefits of bounded (SKRBF, MKRBF, MSRBF) vs. unbounded activation functions (BP). The SKRBF allowed the investigation of kernels with single (SKRBF) vs. multiple width (MKRBF, MSRBF), while the MKRBF tested specifically the incorporation of global (MKRBF) and local (MSRBF) error in the node selection process. Table S1 provides further insight on the training setup for each method and figure S1 presents the details of the training using a genetic algorithm.

#### Landsat Classification Results

Classification accuracy performance was contrasted between the MSRBF with the three aforementioned benchmark algorithms. Different training sample sizes were tested, from 100 to 400 sample points with a 50 point increment. Each training sample had equal representation from both impervious and non-impervious classes. For every sample point size (e.g. 150 points) 50 different training datasets were randomly created from a much larger pool of samples. For every dataset in the samples pool, each network type was tested 50 times (different architectures/parameters) with the settings shown in Table S1. Performance evaluation took place using a single 600 point testing dataset comprised of randomly selected 300 impervious and 300 non-impervious points. All points used for testing were excluded from participating in any of the training datasets. To facilitate direct comparisons all training points were fed into each algorithm, no portion was excluded for evaluation during training (i.e. the calibration dataset was not split into training and testing).

As mentioned, for each of the 50 training datasets per given training size, 50 different algorithms from every of the four algorithmic types were trained resulting in an optimal algorithmic selection based on the highest overall accuracy on the corresponding training dataset. This process created 50 optimal algorithms for each of the four algorithmic types associated with a given training sample point size. These 50×4 algorithms were simulated on the testing dataset and the maximum and average accuracies were reported.

Fig. 7 displays graphically for different training sample point sizes the maximum (hollow bar) and the average (filled bar) values of the best testing accuracy for the MSRBF, BP, SKRBF and MKRBF networks. The MSRBF proposed method outperformed other methods' maximum performance by a margin of 1–2% depending on the benchmark method and the training size. These improvements were more pronounced when contrasting the averages for a 2–3% benefit. Considering the difficulty of the classification task since any pixels with minimal impervious cover are included in the impervious class, this improvement is substantial from the application perspective. Furthermore, analysis provided in Table 1 demonstrates that in every training size scenario the mean accuracy of MSRBF was significantly larger than that of the other algorithms. It is also important to note that higher improvement margins were observed in smaller training dataset sizes. As training data acquisition is always an issue for remote sensing applications, the MSRBF method could find fruitful ground in this field. Furthermore, the standard deviation (T type overlay in Fig. 7) showed higher consistency for the MSRBF. The cumulative standard deviation for all training sample sizes was 1.01% for the MSRBF, 1.13% for the MKRBF, 1.21% for the RBF and 1.28% for the BP networks, respectively.

Of particular interest is the comparison between MSRBF and MKRBF networks. They are RBF type networks, they both support activation functions of variable width and they were both trained with a similar GA-based method. The significant difference is that the MSRBF incorporates local statistics in the node evaluation and adds a blocking layer. The reported assessment indicated that there is a clear benefit associated with the inclusion of local behavior in node evaluation. Another conclusion was that for smaller training point sample sizes the SKRBF slightly outperformed the MKRBF, suggesting that multi-scale RBF networks should be used with caution when sample sizes are small. This could also be attributed to the SKRBF's ability to select any centers for the AFs, while the MKRBF (and the MSRBF) was constrained to center AFs exclusively on training sample point location. The BP networks proved to be a more consistent competitor than the other two benchmarks (MKRBF, SKRBF), possibly due to their unbounded AFs.

### Regression example using waveform LiDAR data

Light Detection And Ranging (LiDAR) data have significantly increased monitoring capabilities due to their ability to extract vertical information [53], [54]. Neural networks have been implemented in several LiDAR studies, for example a BP neural network was constructed to model the tidal terrain using bathymetric LiDAR [55] and a Kohonen Self-Organizing Map was applied in classifying rocks from metrics extracted from airborne LiDAR [56]. The latest LiDAR technology supports a waveform signal return which captures a significantly higher vertical detail by substituting the one to five typical returns points with 200–500 points. Neural network applications in waveform LiDAR are currently limited. Both single layer and multi-layer neural networks were reported in simulated coastal LiDAR waveforms [57], [58]. The simulation results were used in classifying the milt content in the water. It is indicated that the two kinds of neural network showed similar classification results.

#### LiDAR Data

In this experiment, data from the Laser Vegetation Imaging Sensor (LVIS) was used. LVIS is a large footprint airborne waveform LiDAR system designed by NASA with more than 400 returns. The footprint size can vary from 1m to 80 m according to flight height. In this research, the footprint size is nominally 20 m. The laser pulse generated from the sensor has a Gaussian shape both temporally and spatially and operated at a wavelength of 1064 nm [59]. A geolocated waveform of LVIS records reflected pulse from ground objects at a 0.3m vertical resolution. LVIS data has been used for extracting vegetation vertical structure [60], [61]. In this experiment, a total of 162 waveforms were studied from date acquired in Central New York. Field work allowed categorization of these waveforms into five different successional stages [62], [63]: grass, shrub, early stages (3–10 years) and intermediate stages (10 to more than 40 years, split further into coniferous and deciduous).

#### LiDAR Experimental Setup

MSRBF and MKRBF were tested in this experiment. As the dataset in this experiment was limited to waveform returns of which amplitudes were larger than the background noise, a genetic optimization was not applied and the maximum number of nodes was limited to seven. The background noise was estimated by the maximum amplitudes of first 150 waveform returns. Activation function was set to Gaussian function, which contained 3 parameters (i.e. center, amplitude, and width). A node candidate database was created based on the dataset: centers and amplitudes of the candidates were the locations and amplitudes of the returns; 50 widths were evenly distributed within a range between 0 and 1/6 of the time length of the dataset. In MSRBF, no additional condition was set for node selection (e.g. no limit was imposed on the number of sampled points within the local receptive field of an AF). Therefore, the equation for finding the best node only considered balancing global and local accuracy of the AFs (equation 2, R = 0). In order to capture sufficient portion of the signal the weight equation was adapted as following:
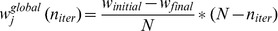
(10)where N is the max number of nodes; 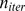 is the current iteration number (i.e. node number). Usually, the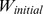 is larger than 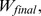 since a large weight is needed at the start of the algorithm(small 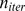 values) to address the large variability within a waveform. The blocking function, where necessary, was set to Y = 0. The, *W-* 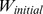
was determined by an exhaustive search using values from 1 to 0 at 0.1 interval for each waveform signal.

Algorithmic evaluation took place using the Mean Absolute Error (MAE) and the Standard Deviation of Errors (SDE). The MAE can evaluate the average performance for an algorithm; however, an algorithm may not perform evenly throughout entire waveform. To receive a more complete evaluation, the SDE was included in evaluating the consistency of performance for each algorithm. Larger SDE in a simulation meant an algorithm performed unequally in different parts of a waveform; while small SDE denoted that the performance was evenly distributed. Good SDE performance is of particular interest to this curve fitting task because high errors can be interpreted as a result of different physical representation (e.g. detect undercanopy where it may not exist).

In order to assess the capability of absorbing variance within a waveform, simulated results from both MSRBF and MKRBF were acquired and normalized with respect to the amplitude of the signal. Thus, the relative MAE and relative SDE were calculated as follows:
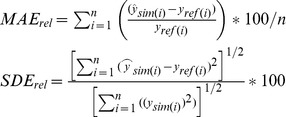
(11)where *i* is one of the point returns forming the waveform; *n* is the total number of return points in a waveform, and 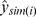 is the simulated amplitude for each return from MSRBF or MKRBF and 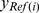
is the reference amplitude for return point *i*. It is important that both MAE and SDE values are low as it would be an indication of a good and consistent fit, respectively.

The purpose of model fitting such waveform LiDAR signals is to extract meaningful and accurate statistics for further processing (e.g. tree heights). In order to do so all waveform points are typically used therefore no cross validation took place. In addition, no BP neural network was compared because the unbounded AFs (e.g. sigmoidal functions) can create significant generalization errors as they do not relate to biophysical parameters as the local bounded functions do (e.g. a Gaussian function relates to undercanopy).

#### LiDAR Regression Results

The comparison results are shown in the Fig. 8. The left column is the MAE comparison and the right column displays SDE results. Each of the four rows corresponds to a different network architecture using 4, 5, 6, or 7 nodes, respectively. Within each of the eight graphs the left side represents results of all plots with further investigation on algorithmic performance in plots of variable signal complexity resulting from different vegetation successional stages. The vertical structure for grass is the simplest within all stages. On the contrary, the intermediate forests, both the deciduous forest and coniferous forest, are more complex since different vertical vegetation layers may exist within a waveform footprint. The five different categories shown are Grass (53 footprints), Shrub (25 footprints), Early Succession (21 footprints), Intermediate Succession Deciduous (25 footprints) and Intermediate Succession Coniferous (38 footprints).

Results indicate that the MSRBF outperforms the MKRBF both in term of minimizing the overall error (MAE) but also in terms of the smaller variability in the fitting errors (SDE). To further investigate these improvements a statistical comparison took place in Table 2, where the improvements were also found statistically significant.

On the surface these improvements, despite their statistical significance, may appear small (e.g. decreasing error by 0.5%). Further analysis was carried out and it is presented in Fig. 9. Three waveforms are presented with the raw signal and the MKRBF and MSRBF simulated curves. Despite the relatively close MAE values, the MSRBF curve is significantly more usable as important biophysical features are preserved. For example, on the top two waveforms the ground is correctly identified only by the MSRBF, while on the bottom waveform the top canopy return is clearly preserved by the MSRBF but not the MKRBF. This is due to the incorporation of local error statistics in the activation function selection process of the MSRBF. On the other hand, the MKRBF only focused on global error minimization therefore returns with large amplitudes dominated the evaluation process allowing returns with small (but biophysically important) amplitudes to be ignored.

## Discussion

The major novelty of the MSRBF neural network, namely the incorporation of local error in the node selection process, allows signals at multiple scales to be revealed and successfully captured. This has led to both statistically significant and application beneficial results. It is important to note that the MKRBF, the current multi-scale RBF used as a benchmark, could be seen as an MSRBF implementation where the local error is not taken under consideration. Therefore, even in the limited cases where the local error analysis may lead to reduced accuracy, the all-encompassing MSRBF framework could dynamically adapt and simply choose to incorporate only global error assessment in the produced model.

Several works have studied optimization techniques (e.g. [64]) and our method could easily support established efficient training methods [65], [66]. Other multi-scale RBF networks have been identified but they lack the ability to handle the overlapping AF issue (see Fig. 1). For example, [67] proposed a hierarchical RBF that dynamically segments the input space in local regions based on a grid structure while the proposed MSRBF does not require a grid and has the ability to combine multiple AFs through an iterative training process. In other efforts, similar to the proposed work, a multi-scale RBF network was introduced with kernels of variable widths [68], [69]. However, the presented solution does not offer a mechanism to handle overlapping signals, expected in multi-width kernels, in an manner other than adding nodes and letting a least squares optimization figure out the relative node contribution at each scale. They are similar to the MKRBF benchmark model contrasted in this study.

A further investigation took place to look into training differences between the MSRBF and the MKRBF networks in the classification scenario. A typical example expressing classification error during training is presented in Fig. 10. Both networks were trained on the same training dataset (300 points) using the same maximum number of nodes (26) and the same pool of candidate AFs. The MKRBF had a 50% classification error (CE) after the introduction of the first node while the MSRBF begun at a much higher value (74%). The initial greediness of the MKRBF continued for the first four nodes; however error absorption saturated after that. On the contrary, the “slow and steady” MSRBF approach compensated for the slow start with further gains at later nodes, reaching a significantly lower global error (10% vs. 22%). Furthermore, the first four nodes had a local CE below the Target Error therefore a blocking process was initiated resulting in 32%, 8%, 10% and 9% absorption of the overall training points for nodes one through four, respectively. A detailed look at the widths of the first MSRBF and MKRBF nodes confirmed the initial MKRBF greediness, since the first MKRBF activation function covered approximately 48% on the entire input space, while the comparable portion for the MSRBF was close to 12%.

In the tested datasets the MSRBF and MKRBF showed similar efficiency in the training process. By design the MSRBF goes through additional calculations as the local error is computed for every node (an average 10% computational cost in the presented examples). However, in most cases the MSRBF required a lower number of nodes to achieve the desired accuracy therefore balancing out the overall computational time to being comparable to the MKRBF. Further optimization techniques could be applied in the future to improve MSRBF's training speed, such as calculation of local errors in a subset of the candidate AFs (e.g. those with sufficient global error absorption).

The generalization ability of the backpropagation neural network was contrasted with the MSRBF by examining the difference between training and testing accuracy. For each training sample point size (100 to 400), the difference between training and testing accuracy was calculated on the 50 optimal networks, as previously described. The mean and standard deviation of this difference is shown in Fig. 11. The MSRBF superiority may be attributed to the fact that the number of sample points within the blocked neighborhood was added as a criterion in the fitness function of the GA algorithm, therefore guiding MSRBF training towards AFs with larger widths.

Back propagation (BP) neural networks are often the first algorithmic choice as opposed to radial basis function (RBF) networks. The major advantage of BP algorithms relates to the fact that node activation functions do not have to be bounded; this allows better generalization ability. On the other hand, RBF networks expect by design bounded activation functions since they are a kernel-based approach. For example, typical BP activation functions are sigmoidal functions, while RBF ones are Gaussian functions. In the presented experiments bounded activation functions were used for all RBF type networks. However, the novel architectural design (see Fig. 4) has the ability to “localize” any function through proper selection of the blocking function in the second layer. Therefore, our network could be seen as a hybrid between RBF and BP type networks, where typical advantages of each network type are preserved, for example the control and transparency of the RBF with the BP large-scale modeling capabilities. Furthermore, by looking at the network architecture of Fig. 4 as a broader integration framework, hidden nodes could be replaced by ancillary models. Different models could be fused together where successfully mapped neighborhoods are assigned to a given model through a selective process that blocks interference from other models. The proposed MSRBF network was discussed in remote sensing tasks but it can easily generalize to classification and regression problems outside this field.

## Supporting Information

Figure S1 Flowchart of genetic algorithm implementation for AF center and width selection.
(TIF)

Table S1 Algorithmic settings for classification assessment.
(DOCX)

Text S1 Adjustment to MSRBF in classification case including the implementation of Genetic algorithm to obtain optimal results.
(DOCX)
