# Supplementary figures and images for: Integrating Local and Global Error Statistics for Multi-Scale RBF Network Training: An Assessment on Remote Sensing Data
Source: PLoS One. 2012 Aug 2;7(8):e40093. doi: 10.1371/journal.pone.0040093 (PMC3411665; doi:10.1371/journal.pone.0040093)

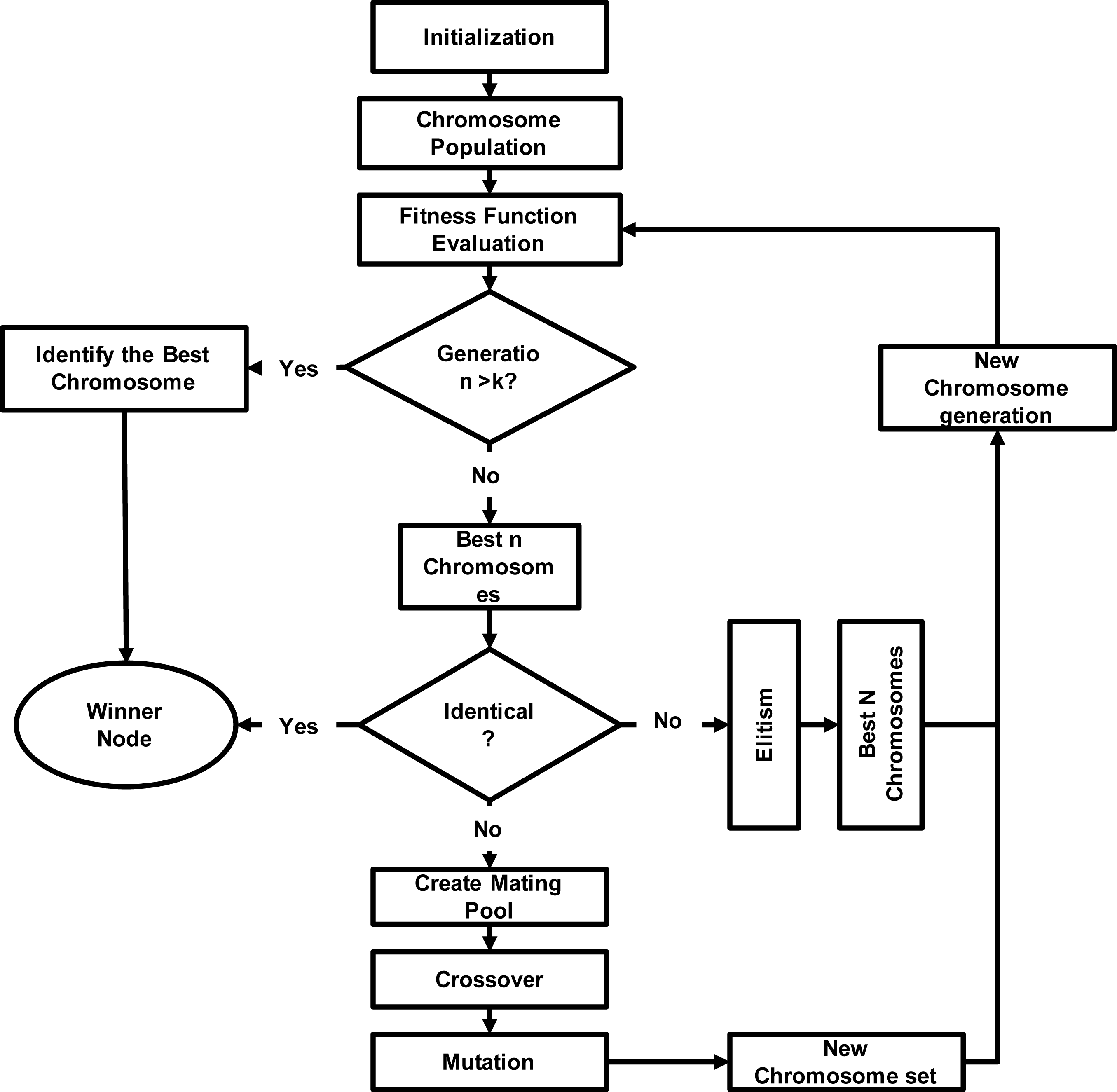

Supplement: Figure S1 — Flowchart of genetic algorithm implementation for AF center and width selection. (TIF) [file pone.0040093.s001.tif]
