# Supplementary material for: Integrating Local and Global Error Statistics for Multi-Scale RBF Network Training: An Assessment on Remote Sensing Data
Source: PLoS One. 2012 Aug 2;7(8):e40093. doi: 10.1371/journal.pone.0040093 (PMC3411665; doi:10.1371/journal.pone.0040093)
Supplement: Table S1 — Algorithmic settings for classification assessment. (DOCX) [file pone.0040093.s002.docx]

**Table S1**. Algorithmic settings for classification assessment

|  | MSRBF (proposed) | BP | MKRBF | SKRBF |
| --- | --- | --- | --- | --- |
| Number of maximum nodes | 20-40 | First layer:1-30 Second layer: 0-15 | 20-40 | 20-40 |
| Learning algorithm | Incremental GA-based learning | Levenberg-Marquardt | Incremental GA-based learning | Orthogonal least squares learning |
| Activation  Function (AF)  Type | Symmetric Sigmoidal  Function | Typical  Sigmoidal Function | Symmetric Sigmoidal Function | Gaussian Function |
| AF centers | From training dataset | N/A | From training dataset | Not constrained |
| AF widths | Multiple | N/A | Multiple | Single |
| Other initialization parameters | Target Error=0.01-0.1;m=1/8-7/8  AF Width=0-1/3 Standard Deviation (SD) of dataset | Goal=0.002  Learning rate= 0.01 | AF Width=0-1/3 SD of dataset | AF Width=A random number within the range of 1/3 SD of dataset |
| Comparison goal | Proposed new method | Well-established method without bounded AFs | Assess incorporation of local statistics and effectiveness of blocking layer | Typical RBF implementation using a single-scale AF |
