# Supplementary material for: Integrating Local and Global Error Statistics for Multi-Scale RBF Network Training: An Assessment on Remote Sensing Data
Source: PLoS One. 2012 Aug 2;7(8):e40093. doi: 10.1371/journal.pone.0040093 (PMC3411665; doi:10.1371/journal.pone.0040093)
Supplement: Text S1 — Adjustment to MSRBF in classification case including the implementation of Genetic algorithm to obtain optimal results. (DOCX) [file pone.0040093.s003.docx]

## **Supporting Information**

## **Materials and Methods**

The text in this section discusses design and implementation details for the classification task.

*Algorithmic Specifics*

In early iterations the local weight is higher to support selection of activation functions (AFs) with good local fits. As the iterations progress, the global classification error (CE) becomes more prominent as the network should start absorbing the overall signal instead of just local portions of it. A blocking function was defined as $B_{j}\left( \vec{x} \right)={-Y}_{c}/\left\{ 1+exp(a_{B}(d_{x}-{width}_{max}) \right\}$ in order to avoid dramatic changes and facilitate to the later classification, when a blocking node was activated. If blocking is initiated for node j, while $B_{j}(\vec{x})=1$ if no blocking is triggered. The blocking function is linked to a specific AF with AF centers described as vector ($\vec{\mu}$), and AF widths ($\vec{s}=[s_{1},s_{2},s_{3}]$ ) positioned in a matrix Sigma = $\left[ \begin{matrix} s_{1}^{2} & 0 & 0 \\ 0 & s_{2}^{2} & 0 \\ 0 & 0 & s_{3}^{2} \end{matrix} \right]$ with ${width}_{max}=max\{s_{1},s_{2},s_{3}\}$. Normalized distance d_x_ is calculated as $d_{x}=\sqrt{K^{T}*{Sigma}^{-1}*K}$, where K= [$\vec{x}$-$\vec{\mu}$]. *Y_c_* is the class value associated with the center point $\vec{\mu}$ , $\vec{x}$is the input vector; and *a_B_* is the predefined slope of the blocking function curve (-10 in our case). Note that the non-diagonal elements of the sigma matrix were zero as no rotations were allowed.

*MSRBF and benchmark setup*

To evaluate the effectiveness of the MSRBF network, a classification accuracy comparison between several algorithms was carried out, namely a Back-Propagation (BP), a single kernel RBF (SKRBF) and a multi-kernel RBF (MKRBF) neural networks. The SKRBF was based on the built-in Matlab code and employed the same width for all kernel functions. The BP also used Matlab’s built-in functions with the default Levenberg-Marquardt algorithm. The MKRBF was custom coded with similar properties as the proposed MSRBF. Table S1 provides further insight on the training setup for each method.

*Genetic Algorithm implementation in selecting nodes for classification problem*

The MSRBF training had three goals, progressively moving from left to right in Fig. 4: activation function (AF) identification for every node (centers and widths), identification of a blocking index and identification of network weights. The AF properties were selected in an iterative process using a Genetic Algorithm (GA) for incremental learning. The GA is a global heuristic searching technique that finds the optimal or near optimal solution [[70](#_ENREF_70)]. GA approaches are frequently utilized in various applications of remote sensing for deriving optimal parameters for a specific model [[71](#_ENREF_71),[72](#_ENREF_72),[73](#_ENREF_73),[74](#_ENREF_74)]. In the training process of MSRBF, the GA was applied on the identification of the winning node.

Before training a predetermined classification error was defined as Target Error that expressed a successful simulation. Also, a maximum number of hidden layer nodes were provided. At all iterations the following process took place to select the winning activation function (AF) from all candidates:

1. Calculate the global classification error (CE) for each AF.
2. If the global CE is less than a predefined threshold accept that AF as the winning node and stop adding further nodes.
3. Calculate the local classification error (CE) for each AF.
4. Optimize AF parameters using a GA approach and the integrated local/global error criterion.
5. Repeat until the maximum number of hidden layer nodes is reached.

At the beginning of the algorithm (Fig. S1), a chromosome population was randomly generated with the same size as the training dataset. In the population, the real-coded chromosome had six genes, the first three genes were associated with the AF centers and the other three provided AF widths for the three different dimensions, respectively. The center chromosomes were restricted to taking values from existing points in the training dataset, while the width chromosomes were allowed to take random values within 1/3 of the standard deviation of the training dataset in each dimension. All chromosomes were evaluated by the fitness function, which was the weighted sum of the global CE and the local CE shown in equation (8). After the fitness function evaluation all chromosomes were ranked. If the generation number was equal to the max generation number (k=20 in our experiment), the best chromosome was selected providing the AF width and center parameters for the winner node; otherwise, the best n (n=10 in our experiment) chromosomes were extracted for further examination. If all best chromosomes were identical then that chromosome would be the winner node and the process would exit. If not, an elitism step was activated to pass these best chromosomes to the next generation. The remaining chromosomes of the next generation were created by a roulette wheel role. All but the best n chromosomes were forwarded to a mating pool. Chromosomes with higher fitness had a higher probability of selection for the crossover and mutation processes to create a new chromosome set.

After the hidden layer nodes were identified (including number of nodes and AF parameters for each node) an index for the blocking layer was created. This index acts as a binary filter blocking subsequent node influence from a local neighborhood. It is initiated when that local neighborhood has been successfully mapped, in order words when the local CE of that node is less than the Target Error. Since this information is already calculated in the GA process, this index is easily created.

At the final step, MSRBF weights are identified through a least squares solution using a pseudoinverse process to avoid singularity issues. Typically, weights of blocked AFs are close to 1, while other weights express minor adjustments, especially if nodes having AFs with close centers are identified.

70. Goldberg DE (2008) Genetic Algorithms in Search, Optimization, and Machine Learning. Reading, MA: Addison-Wesley.

71. Chion C, Landry JA, Da Costa L (2008) A genetic-programming-based method for hyperspectral data information extraction: Agricultural applications. IEEE Transactions on Geoscience and Remote Sensing 46: 2446-2457.

72. Ghoggali N, Melgani F, Bazi Y (2009) A multiobjective genetic SVM approach for classification problems with limited training samples. IEEE Transactions on Geoscience and Remote Sensing 47: 1707-1718.

73. Shan J, Alkheder S, Wang J (2008) Genetic algorithms for the calibration of cellular automata urban growth modeling. Photogrammetric Engineering and Remote Sensing 74: 1267-1277.

74. Stathakis D (2009) How many hidden layers and nodes? International Journal of Remote Sensing 30: 2133-2147.
